# Proteomic Analysis of *Trichinella spiralis* Muscle Larval Excretory-Secretory Proteins Recognized by Early Infection Sera

**DOI:** 10.1155/2013/139745

**Published:** 2013-06-13

**Authors:** Li Wang, Zhong Quan Wang, Dan Dan Hu, Jing Cui

**Affiliations:** Department of Parasitology, Medical College, Zhengzhou University, 40 Daxue Road, Zhengzhou 450052, China

## Abstract

Although the excretory-secretory (ES) proteins of *Trichinella spiralis* muscle larvae are the most commonly used diagnostic antigens for trichinellosis, their main disadvantage is the false negative results during the early stage of infection and cross-reaction of their main components (43, 45, 49, and 53 kDa) with sera of patients with other helminthiasis. The aim of this study was to identify early specific diagnostic antigens in *T. spiralis* ES proteins with 30–40 kDa. The ES proteins were analyzed by two-dimensional electrophoresis (2-DE), and a total of approximately 150 proteins spots were detected with isoelectric point (pI) varying from 4 to 7 and molecular weight from 14 to 66 kDa. When probed with sera from infected mice at 18 days postinfection, ten protein spots with molecular weight of 30–40 kDa were recognized and identified by MALDI-TOF/TOF-MS. All of ten spots were successfully identified and characterized to correlate with five different proteins, including two potential serine proteases, one antigen targeted by protective antibodies, one deoxyribonuclease (DNase) II, and one conserved hypothetical protein. These proteins might be the early specific diagnostic antigens for trichinellosis.

## 1. Introduction

Trichinellosis remains an important food-borne parasitic zoonosis in many parts of the world [1, 2]. Humans acquire the disease by the ingestion of raw or insufficiently cooked meat containing the infective larvae of the nematode's genus *Trichinella *[3]. In heavy infections, trichinellosis can be fatal. From 2004 to 2009, 15 outbreaks of human trichinellosis, consisting of 1 387 cases and 4 deaths, were reported in China [4]. So, trichinellosis is not only a public health hazard by affecting patients but also represents an economic problem in porcine animal production and food safety.

The diagnosis of trichinellosis is rather difficult because the signs and symptoms are nonspecific [5, 6]. Up to now, a definitive diagnosis of human trichinellosis can be made only by detecting larvae in a muscle biopsy or by highly specific immunodiagnostic tests [7, 8]. The sensitivity of muscle biopsy depends on the amount of muscle sample tested and the degree of infection. Muscle biopsy is not sensitive to the light infections and the early stage of infection. The excretory-secretory (ES) proteins of *T. spiralis *muscle larvae (ML) are the most commonly used diagnostic antigens for trichinellosis [5, 7], but their main disadvantages are the false negative results during the early stage of infection and cross-reaction with other parasites [8, 9]. The ES antigens of* T. spiralis *ML were recommended to be used in Western blot for detecting anti-*Trichinella *antibodies by the International Commission on Trichinellosis (ICT) [10], and their main components were 43, 45, 49, and 53 kDa proteins analyzed by SDS-PAGE or two-dimensional electrophoresis (2-DE) gel analysis [11, 12]. However, these main components of *T. spiralis* ES proteins were usually cross-reacted with sera of patients with echinococcosis, cysticercosis, schistosomiasis, paragonimiasis, and clonorchiasis [9, 13–15]. This is particularly important in developing countries where human helminth infections are common and cross-reactions with these parasites could give false positive results [16]. Although the risk of cross-reactions using ES proteins is low in industrialized countries, cross-reactions do occur with anisakiasis or other larval migrans of unknown species. An ELISA method using purified tyvelose-containing antigen from muscle larvae of *T. spiralis* is sensitive and specific for immunodiagnosis of trichinellosis, but it is not useful for early diagnosis [9]. Hence, there is an urgent need to develop the new specific antigens for early diagnosis of trichinellosis. 

Immunoproteomics, a technique involving 2-DE, followed by immunoblotting, is an approach to identify specific antigenic proteins in high resolution in a wide range of proteins expressed by different organisms [17]. Identification of the immunogenic proteins will help us to screen novel serological diagnostic markers and vaccine candidates. However, the parasitic soluble or ES proteins were often screened by immunoblotting using the immune sera or sera at 4–6 weeks after infection, and it is difficult to identify the early antigens [18–20]. To our knowledge, no early antigens of *T. spiralis *larvae have been immunoblotted and identified by tandem mass spectrometry.

The purpose of this study was to identify the early specific diagnostic antigens in *T. spiralis* ES proteins recognized by early infection sera. The ES proteins from ML were analyzed by 2-DE and Western blot probed with early infection sera at 18 days postinfection (DPI). Then, the main immunoreactive protein spots with 30–40 kDa were identified and characterized by Matrix-assisted-laser-desorption-ionization- (MALDI-) time-of-flight (TOF)/TOF-MS analyses in combination with bioinformatics analysis.

## 2. Materials and Methods

### 2.1. Parasite and Experimental Animals

*Trichinella spiralis *isolate (ISS534) used in this study was obtained from a domestic pig in Nanyang city of Henan province, China. The isolate was maintained by serial passages in Kunming mice in our laboratory. Muscle larvae were recovered from the mice infected with *T. spiralis* at 42 DPI by artificial digestion as described previously [21, 22]. Female specific pathogen free (SPF) BALB/c mice aged 6 weeks were purchased from the Experimental Animal Center of Henan province (Zhengzhou, China). The permission (no. SCXK 2010-0002) was given by the Science and Technology Department of Henan Province. All procedures of animal experiment of this study were approved by the Life Science Ethics Committee of Zhengzhou University.

### 2.2. Collection of Sera

Forty BALB/c mice were orally infected with 300 ML/mouse and the serum samples from the infected mice were collected as described previously [23]. About 100 *μ*L of tail vein blood was daily collected from each mouse before infection and during 14–21 DPI, respectively. When the forty infected mice were sacrificed at 42 DPI by deep ether anesthesia, their serum samples were also collected. 

### 2.3. Preparation of ES Proteins

Preparation of ES proteins was performed as previously described [24, 25]. Briefly, after washing thoroughly in sterile saline and serum-free RPMI-1640 medium supplemented with 100 U penicillin/mL and 100 *μ*g streptomycin/mL, the larvae were incubated in the same medium at 5000 worms/mL for 18 h at 37°C in 5% CO_2_. After incubation, the media-contained ES proteins were poured into 50-mL conical tubes and the larvae were allowed to settle for 20 min. The supernatant containing the ES products was filtered through a 0.2 *μ*m membrane. The ES products were dialyzed and then lyophilized by a vacuum concentration and freeze-drying (Heto Mxi-Dry-Lyo, Denmark). The protein concentration was determined by the Bradford assay [26]. 

### 2.4. 2-DE

The ES proteins were precipitated using trichloroacetic acid (TCA) and acetone as previously described method with some modifications [27]. Briefly, the sample was suspended in 10% TCA in acetone with 20 mM DTT for 2 h at −20°C. After centrifugation at 15 000 g for 15 min at 4°C, the pellet was resuspended in cold acetone containing 20 mM DTT and washed for three times. The final pellet was air-dried. The 2-D electrophoresis was performed as previously described [28]. Briefly, the pellet was suspended in rehydration buffer (7 M urea, 2 M thiourea, 4% CHAPS, 65 mM DTT, 0.2% IPG buffer (pH 3–10), and 0.001% bromophenol blue), containing 800 *μ*g of the protein sample in a total volume of 500 *μ*L and centrifuged at 12 000 g for 10 min at room temperature to remove the insoluble materials. The supernatant was loaded onto 24 cm pH 3–10 immobilized pH gradient (IPG) strips (Bio-Rad, USA) by overnight reswelling and separated by isoelectric focusing (IEF) using a Protean IEF Cell (Bio-Rad, USA). IEF was performed using a Protean IEF Cell at 20°C as follows: S1: 250 V, 30 min; S2: 500 V, 30 min; S3: 1000 V, 1 h; S4: 10 000 V, 5 h; and S5: 10 000 V, 60 000 Vh (using a limit of 50 *μ*A/strip). After IEF, the IPG strips were equilibrated sequentially, first in equilibration buffer (6 M urea, 0.375 M Tris-HCl pH 8.8, 2% SDS, and 20% glycerol) containing 2% dithiothreitol, then in equilibration buffer containing 2.5% iodoacetamide. The second dimension was performed on 12% SDS-PAGE using a Mini Protean cell (Bio-Rad, USA). Proteins were separated for 30 min at 16 mA and then at 24 mA until the dye front reached the bottom of the gel at 16°C. Three replicates were run for the sample. After 2D gel electrophoresis, proteins were either stained with Coomassie blue R-250 for proteomic analysis or used for immunoblotting as previously described [29]. The tests were repeated three times.

### 2.5. Western Blot Analysis

Proteins from 2-DE gels were transferred onto polyvinylidene difluoride membrane (PVDF) membranes by semidry transfer cell (Bio-Rad, USA) for 1 h at 20 V [24]. The membranes were blocked with 5% skimmed milk for 2 h at room temperature. Following three washes with TBST, the membranes were incubated overnight at 4°C with sera of infected mice (1 : 100 dilution). After washing with TBST, blots were then incubated with horseradish-peroxidase- (HRP-) conjugated goat anti-mouse IgG (Sigma, USA) (1: 5,000 dilution) at 37°C for 1 h. Following three additional washes, the membranes were developed with the enhanced chemiluminescent (ECL) kit (CWBIO, China). Sera collected from mice before infection were used as controls. Immunoblot experiments were conducted in duplicate, with no variation in results observed. Images of immunoblots were captured using ImageScanner (GE healthcare, USA) and aligned with equivalent protein stained 2-DE gels using Image Master 2D Platinum 6.0 (GE healthcare, USA).

### 2.6. 2-DE Gel Excision and Tryptic Digestion

2-DE gel electrophoresis protein spots with low molecular weight recognized by early infection sera were prepared for MALDI-TOF/TOF-MS analysis according to standard protocols [30]. Ten immunoreactive spots with low molecular weight were excised manually from the Coomassie blue-stained gels. The excised gel pieces carrying the spots were placed in a tube, destained for 20 min in 200 mmol/L NH_4_HCO_3_/30% acetonitrile, and then lyophilized. All the lyophilized samples were digested overnight at 37°C with 12.5 ng/mL trypsin in 25 mmol/L NH_4_HCO_3_. The peptides were extracted three times with 60% ACN/0.1% trifluoroacetic acid (TFA). The extracts were pooled and dried completely by centrifugal lyophilization.

### 2.7. Protein Identification by MALDI-TOF/TOF-MS

The resulting peptides from the above extraction were analyzed by MALDI-TOF/TOF-MS. The procedure was performed as described previously [31]. Briefly, The purified tryptic peptide samples were spotted onto stainless steel sample target plates and mixed (1 : 1 ratio) with a matrix consisting of a saturated solution of a-cyano-4-hydroxy-trans-cinnamic acid in 50% acetonitrile-1% TFA. Peptide mass spectra were obtained on an Applied Biosystem Sciex 4800 MALDI-TOF/TOF mass spectrometer (Applied Biosystems, USA). Data were acquired using a CalMix5 standard to calibrate the instrument (ABI4700 Calibration Mixture). The MS spectra were recorded in reflector mode in a mass range from 800 to 4000 with a focus mass of 2000. For MS/MS spectra, up to 10 of the most abundant precursor ions per sample were selected as precursors for MS/MS acquisition, excluding the trypsin autolysis peaks and the matrix ion signals. In MS/MS positive ion mode, for one main MS spectrum, 50 subspectra with 50 shots per subspectrum were accumulated using a random search pattern. Collision energy was 2 kV, collision gas was air, and default calibration was set by using the Glu1-Fibrino-peptide B ([M+H] + 1, 570.6696) spotted onto Cal 7 positions of the MALDI target. Combined peptide mass fingerprinting (PMF) and MS/MS queries were performed by using the MASCOT search engine 2.2 (Matrix Science, UK) and submitted to MASCOT Sequence Query server (http://www.matrixscience.com) for identification against nonredundant NCBI database (http://www.ncbi.nlm.nih.gov/BLAST) with the following parameter settings: 100 ppm mass accuracy, trypsin cleavage (one missed cleavage allowed), carbamidomethylation set as fixed modification, oxidation of methionine allowed as variable modification, and MS/MS fragment tolerance set to 0.4 Da. The criteria for successfully identified proteins were as follows: ion score confidence index (CI) for peptide mass fingerprint and MS/MS data was ≥95%.

## 3. Results

### 3.1. 2-DE Analysis of *T. spiralis *ES Proteins

**Figure 1:**
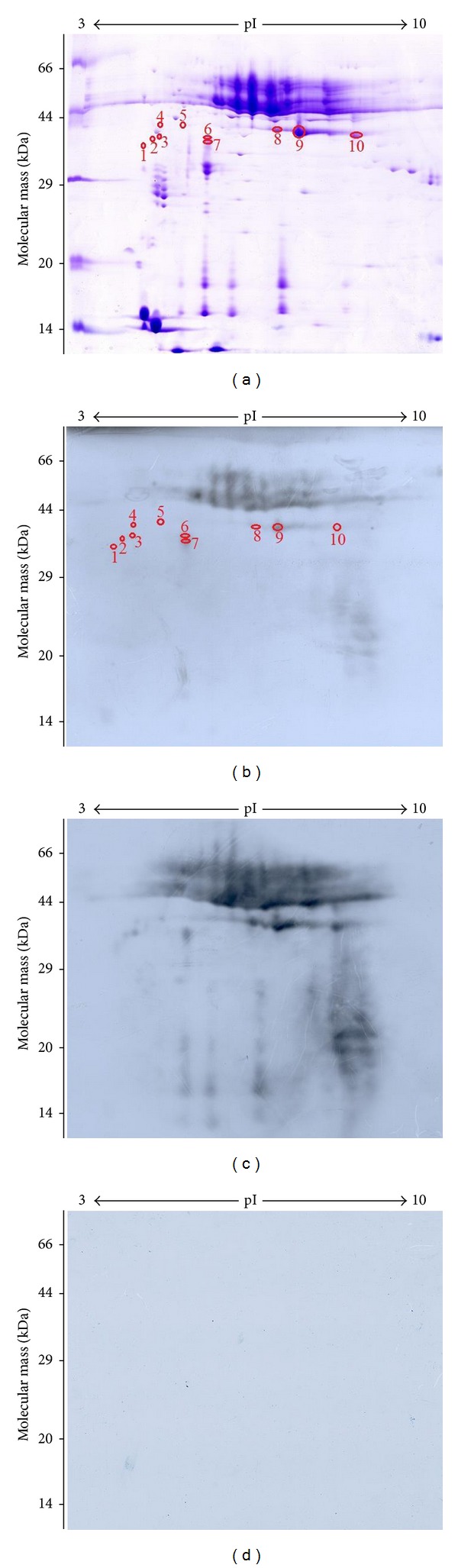
2-DE and Western blot analysis of *T. spiralis *muscle larval excretory-secretory (ES) proteins. (a) 2-DE gel of ES proteins separated in the first dimension in the pH range 3–10 and then in the second dimension on a 12% polyacrylamide gel. The gel was stained with Coomassie blue R-250, molecular weight standard is on the left, and pI values are indicated. Protein spots selected for analysis are numbered. (b) 2-DE Western blot of ES proteins probed by mouse infection sera at 18 days postinfection (DPI), and the immunoreactive protein spots were detected by the enhanced chemiluminescent (ECL) kit. (c) 2-DE Western blot of ES proteins probed by mouse infection sera at 42 DPI. (d) Western blot map probed by sera from mice before infection.

The ES proteins of *T. spiralis *ML were separated on a 2-DE gel covering a pH 3–10 nonlinear, and the protein spots were visualized following Coomasie R-250 staining (Figure 1(a)). A total of approximately 150 spots were detected on the Coomassie bule stained 2-DE gels, with pI varying from 4 to 7 and molecular weight from 14 to 66 kDa. Major protein spots were located in acidic range (pH 4–7) migrating at 30–60 kDa. The 2-DE was repeated three times, and the patterns were highly reproducible. 

### 3.2. Western Blot Analysis of *T. spiralis *ES Proteins following 2-DE

**Figure 2:**
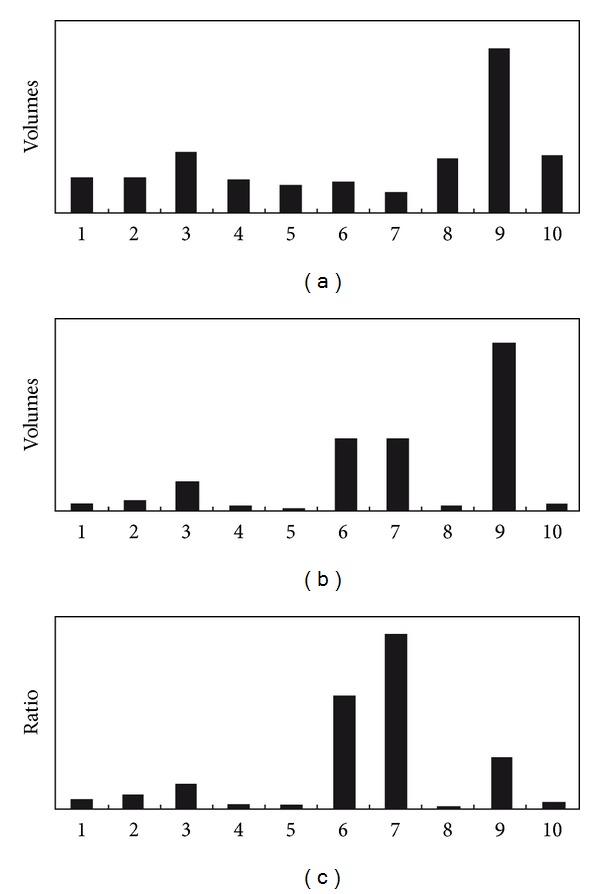
Volumes of *T. spiralis *ES protein scanning spots of 1–10 from 2-DE and 2-DE Western blot and the comparison of their ratios. (a) Volumes of scanning spots from 2-DE. (b) Volume of scanning spots from 2-DE Western blot. (c) Ratios between respective scanning spots on 2-DE Western blot and 2-DE.

Anti-*Trichinella *IgG antibodies in sera from infected mice at 14–21 DPI were assayed by ELISA and Western blot. The specific antibodies were firstly detected at 18 DPI by the above-mentioned two methods (data not shown), and then these sera were used to detect the fractions of ES proteins. As shown in Figure 1(b), there were more than 30 spots displaying reactivity to the infection sera at 18 DPI. Once photographed, the immunoblot and their homologous Coomassie blue-stained gel were aligned and then matched by Image Master 2D Platinum 6.0 software and artificial recognition, and 25 of those positive spots were found to be precisely matched with the homologous gel. Out of the 25 matched positive spots, 10 spots named spot 1 to 10 with 30–40 kDa were selected to be further analyzed by MS. Interestingly, we found that the volumes of spots of 1–10 were not proportional between 2-DE and Western blot. Spot 9 in 2-DE and Western blot showed the highest volumes. Therefore, comparisons of their ratios may show differences in immunogenicity. The activities of the immunogens ordered from highest to lowest are as follows: 7, 6, 9, 3, 2, 1, 10, 5, 4, and 8 (Figure 2). In comparison, when the immunoblot was performed with sera at 42 DPI, there were additional 30 positive spots (Figure 1(c)) apart from the above-mentioned 25 spots, indicating that the 25 positive spots were recognized by both of sera at 18 DPI and 42 DPI, but the stronger reactions were observed with sera at 42 DPI, and the additional 30 positive spots were recognized only by sera at 42 DPI. But the sera collected from mice before infection did not show detectable immunoreactivity with any of the protein spots (Figure 1(d)). 

### 3.3. Identification of Immunoreactive Proteins by MALDI-TOF/TOF-MS

All 10 matched protein spots with 30–40 kDa recognized by sera at 18 DPI were successfully identified and characterized to correlate with five different proteins, including two potential serine proteases with different pI and molecular weight values, one antigen targeted by protective antibodies, one deoxyribonuclease (DNase) II, and one conserved hypothetical protein. The results of protein identification are shown in Table 1.

### 3.4. Functional Categorization of Immunoreactive Proteins by Gene Ontology

To further understand the functions of immunoreactive proteins with 30–40 kDa recognized by early infection sera, Gene Ontology Annotation was performed. The annotated *T. spiralis *proteins from the NCBI nonredundant database were analyzed by the on-line Blast2GO tool. Each identified known protein was classified according to its GO functional annotation. For the molecular function ontology, the proteins are related to peptidase activity acting on L-amino acid peptides (4, 44.4%), serine-type peptidase activity (4, 44.4%), and nuclease activity (1, 11.1%). On the biological process category, the five proteins were essentially those involved in metabolism of proteins (4, 57.1%), nucleoside, nucleotide and nucleic acid (1, 14.3%), cellular macromolecule (1, 14.3%), and nitrogen compound (1, 14.3%), which might play an important role in the development of *T. spiralis *larvae in the host. As for the cellular component ontology, the proteins are assigned as cell part proteins (4, 100%). In the subcategory of cell part, two were ascribed to membrane, and the other two were assigned to membrane part, respectively, suggesting that these membrane proteins may be the main early target antigens.

## 4. Discussions

Modulation of the immune response by helminthes involves the ES proteins released by these parasites. These proteins include proteases, protease inhibitors, allergen homologues, glycolytic enzymes, lipids, and glycans, which together or individually could be potential immunomodulators [32, 33]. ES proteins of *T. spiralis* ML come mainly from the excretory granules of the stichosome and the cuticles (membrane proteins) and are directly exposed to the host's immune system and are the main target antigens which induce the immune responses [34]. In this study, our results also demonstrated a protein pattern of the *T. spiralis *ES proteins migrating between 40 and 60 kDa (as shown Figure 1(a)). Since these main components (43, 45, 49, and 53 kDa) of *T. spiralis* ES proteins were usually cross-reacted with sera of patients with other helminthiasis, ten ES protein spots with molecular weight of 30–40 kDa recognized by sera at 18 DPI were selected to be further identified by mass spectrometry.

In recent years, the immunoproteomic approach has been successfully applied to analyze both the soluble and ES proteins of *Trichinella *ML probed with infection sera at 4–6 weeks after infection, immune sera, or monoclonal antibody [18, 19, 28, 31]. In this study, an attempt is made to screen early specific antigens from *T. spiralis *ES proteins, which might be useful for the early diagnosis of trichinellosis. Our results showed that 10 protein spots with 30–40 kDa were recognized by sera at 18 DPI and successfully identified by MALDI-TOF/TOF-MS. The 10 protein spots identified represented only five different proteins: four matched known *T. spiralis *proteins and the remaining one to conserved hypothetical protein, and all of them had protease activities. Protease activities have been identified in ES proteins of *T. spiralis*. Moreover, proteinase can serve as an immunodominant antigen, stimulating a protective immune response. A previous study has also demonstrated that *T. spiralis *may express more than one isoforms of the protein and that a common precursor protein could undergo variable posttranslational processing [35]. In this study, DNAase II, antigen targeted by protective antibodies and serine proteases are all identified in multiple protein spots. DNase II is known to be a lysosomal enzyme, introduces single and double-stranded breaks into supercoiled plasmids in the presence of EDTA, and mediates internucleosomal DNA digestion characteristic of apoptosis following intracellular acidification [36, 37]. Four spots (1, 3, 4, and 5) were identified as DNase II. Interestingly, spot 3 and 4 have the same pI but different MW. On the contrary, spots 4 and 5 have the same MW but different pI. But spot 1 has the lower pI and MW than spots 3, 4, and 5. In addition, two spots (6 and 7) were identified as antigen targeted by protective antibodies. In comparison, they have the same pI but small differences in MW. These spots (1, 3, 4, and 5; 6 and 7) may have different isoforms of the same protein, as a result of alternative splicing, posttranslational modifications, and protein processing [18, 31]. These modifications could be related to phosphorylation or acetylation of the proteins after translation, and they could be vital for the protein's biological functions, such as parasite survival, immune escape, and immunopathogenesis. Besides, the observations that three spots (8, 9, and 10) are matched to TspSP-1, support previous data that there are multiple isoforms of this protein present in the ES fraction [38]. Comparison against the amino acid sequence of SP-1.2 and SP-1.3 was performed using the NCBI-BLAST (http://www.ncbi.nlm.nih.gov/BLAST) network server, and the amino acid sequence similarity between them was 84%. In addition, both of the conserved hypothetical protein (gi*|*316966524) and the antigen targeted by protective antibodies (gi*|*404638) belong to the trypsin-like serine protease (Tryp_SPc) superfamily, and the amino acid sequence similarity between them was up to 90%. The above-mentioned five proteins from *T. spiralis *ES proteins recognized by early infection sera might be the early specific diagnostic antigens for trichinellosis, which is needed to be further confirmed in the experiments.

## Figures and Tables

**Table 1:** Identification of *T. spiralis *ES proteins with 30–40 kDa recognized by mouse infection sera at 18 DPI using MALDI-TOF/TOF-MS.

Spot no.	Protein name	Accession Number	Theoretical pI/Mr	MOWSE score	Coverage (%)	Number matched peptides	*P* value
1	Deoxyribonuclease II	gi∣316974621	5.95/38.1	151	12	7	2.6*e* − 011
2	Conserved hypothetical protein	gi∣316966524	4.85/28.5	118	8	2	5.3*e* − 008
3	Deoxyribonuclease II	gi∣316974621	5.95/38.1	169	16	5	4.2*e* − 013
4	Deoxyribonuclease II	gi∣316974621	5.95/38.1	239	25	7	4.2*e* − 020
5	Deoxyribonuclease II	gi∣316974621	5.95/38.1	404	29	7	1.3*e* − 036
6	Antigen targeted by protective antibodies	gi∣404638	4.76/31.7	292	30	6	2.1*e* − 025
7	Antigen targeted by protective antibodies	gi∣404638	4.76/31.7	354	30	6	1.3*e* − 031
8	Serine protease	gi∣168805931	5.97/35.7	470	30	8	3.3*e* − 043
9	Serine protease	gi∣168805931	5.97/35.7	291	29	6	2.6*e* − 025
10	Serine protease	gi∣168805933	6.33/48.7	425	24	9	1.1*e* − 038
